# Revisiting the Concept of Vigilance

**DOI:** 10.3389/fpsyt.2022.874757

**Published:** 2022-06-14

**Authors:** Gerhard Klösch, Josef Zeitlhofer, Osman Ipsiroglu

**Affiliations:** ^1^Department of Neurology, Sleep Lab, Medical University of Vienna, Vienna, Austria; ^2^Institute for Sleep-Wake-Research, Vienna, Austria; ^3^Faculty of Psychotherapy Science, Sigmund Freud Private University, Vienna, Austria; ^4^Department of Pediatrics, Faculty of Medicine, University of British Columbia, Vancouver, BC, Canada; ^5^H-Behaviours Research Lab, BC Children's Hospital Research Institute, University of British Columbia, Vancouver, BC, Canada

**Keywords:** vigilance, sleep-wake behaviors, children sleep disorders, daytime sleepiness, alertness

## Abstract

Vigilance deficits can be observed after a period of prolonged, continuous wakefulness. In this context there has been extensive research targeting the impact of sleep deficits on different aspects of vigilance, but the underlying concept of vigilance was hardly ever addressed and discussed. One reason for this shortcoming is the unclear and ambiguous definition of the term vigilance, which is commonly used interchangeably with sustained attention and even wakefulness. This confusion is the result of a wide range of misleading definitions, starting in the 1940s, as psychologists redefined the concept of vigilance suggested by British Neurologist, Henry Head, in 1923. Nevertheless, the concept of vigilance is still useful and innovative, especially in treating sleep problems in children and young adults. This paper reviews the current usage of the term vigilance in sleep-wake-research and describes not only the benefits, but even more clearly, its limitations. By re-focusing on the definitions given by Henry Head, the concept of vigilance is an innovative way to gather new insights into the interplay between sleep– and daytime behaviors. In addition, future research on vigilance should consider three perspectives: 1st vigilance perceived as a process to allocate resources, 2nd vigilance associated with compensatory behaviors and 3rd the role of vigilance in human environmental interactions. This approach, understood as a conceptual framework, provides new perspectives by targeting sleep-wake behaviors as a ‘real life’ outcome measure, reflecting both physical and cognitive performance as well as sleep quality and quantity.

## The Many Facets of the Construct Vigilance

Hardly any other concept has caused as much confusion with its definition in psychology, physiology, and sleep research than the term vigilance. In everyday language, vigilance (derived from the Latin word, *vigilantia*) is primarily associated with *being highly alert* or having *sustained attention*. In the scientific context, the term *vigilia* has long been used to describe sleeplessness, but in current usage, the psychological definition as a state of *increased and longer-lasting responsiveness* has prevailed. These approaches have little to do with the concept of vigilance, as suggested in 1923 by Henry Head, a British neurologist (1). He referred to vigilance as the organism's ability to reorganize itself and restore damaged functions. After trauma, the first signs of “vigilance” were the reappearance of reflexes, followed by automatic actions and gestures, and finally the regaining of the ability to differentiate between sensory stimuli (readiness to respond). In Head's opinion, the reappearance of sensory processing is purely physiological in nature and independent of higher cognitive functions such as consciousness, motivation or interest. Therefore, vigilance is neither a cognitive skill nor a matter of consciousness. However, consciousness requires vigilance and adequate processing of sensory inputs or a functioning autonomic nervous system (e.g., to control blood pressure, body temperature, etc.). In this context, vigilance is a universal property of animals and humans in order to react adequately to environmental stimuli and to ensure the survival of the individual.

Head defined three sub-categories as essential for his concept of vigilance: 1st *perception*, to guarantee that a stimulus is registered by the sensory system according to their modality. 2nd *behaviors*, a category which includes all kinds of observable behavior, whereby complex behavior is associated with higher levels of vigilance (and vice versa). And 3rd *reorganization*, referring to the ability of injured organisms to reorganize and restructure their neuronal connections in order to take over or compensate for the function of damaged structures. The goal of reorganization is to ensure the survival of the individual, which is by far the key function of vigilance (1).

Nevertheless, the reorganization aspect of vigilance caused confusion and criticism because it was not entirely clear what it meant. This was partly due to Head's inconsistent use of the term vigilance, which he sometimes referred to as vital energy, in relation to both nervous and mental processes. More than that, it remains unclear whether vigilance is *per se* the reorganizing force or only the result of this process (2).

## The Concept of Vigilance in Experimental Psychology

In the view of test psychologists, vigilance can be measured by a simple S-R model (stimulus-response). This approach assumes that the presentation of a stimulus leads to similar responses in all individuals or to the same class of responses, observed over a distinct period of time. These time-on-task effects are seen particularly in behavioral automatisms. However, the dynamic aspect of Head's concept of vigilance (as a self-organizing system) is not addressed by conventional S-R models. For this purpose, more sophisticated approaches such as dynamic self-regulation models are necessary. Regardless of these possibilities, in the 1940s, NH. Mackworth developed, on behalf of the British Airforce, vigilance tasks (utilizing the Mackworth clock-test) in order to recruit suitable personnel for radar surveillance activities. Systematic studies with the clock-test (lasting for more than 2 h) demonstrate that even highly motivated individuals found it difficult to maintain their attention at a high level for such a long time without making mistakes. Mackworth (3) defined the ability to be attentive over long time periods as vigilance (or sustained attention) and fluctuations in attention as vigilance decrement, which was by far closer to the everyday understanding of vigilance (in the sense of being highly alert) than to Head's conceptual framework.

As a matter of fact, much of the vigilance research was conducted at the beginning of the Cold War in the 1950s, a time when slogans such as “constant vigilance” were common rhetorical figures in political communication in the Western World and the Soviet Union. Under these circumstances, vigilance research became an important discipline and subject of military defense strategies (4). But the military influence on vigilance research was criticized and not commonly accepted in the research community (5). In addition, the requirements for air traffic controllers had changed radically since the time Mackworth developed his test methods. Instead of reacting to rare events in monotonous situations, the increasing frequency in commercial air traffic generates a continuous stream of information and requires other skills such as a high degree of flexibility and the ability to deal simultaneously with different kinds of stimuli. In comparison, the conditions of the Mackworth clock-test are far less complex. Besides the assumption that vigilance tests should mimic detection performance during prolonged watch-standing conditions, the necessity of additional characteristics to classify a vigilance task was evident. Otherwise, vigilance monitoring would not differ from research on simple reaction time, which is still a common view-point (6). Although this issue was excessively discussed, there is still no agreement on the main characteristics of a vigilance task (e.g., test duration, type of stimulus and their temporal order) and obligatory outcome measurements (performance characteristics, response definitions, etc.). Even Mackworth's assumption that vigilance tests should last sufficiently long (e.g., 2 h and more) has become obsolete since the release of the 10 min version of the PVT (7, 8).

The lack of standardization for measuring vigilant performance and their interpretation led to an extensive and unreflective use of otherwise well-established psychometric test procedures such as simple reaction time tests, forced choice– or go/no-go tasks. This opened the door for an increasing number of alternative explanations and terms such as tonic alertness (9) or vigilant attention (10). Regardless of these developments, the Mackworth clock-test is still in use, even in slightly modified and computerized versions (11, 12). And besides its spongy definition, most studies with sleep deprived subjects refer to the concept of vigilance to describe the significant impairments caused by less or inadequate sleep (10, 13, 14).

Reconsidering Head's concept of vigilance, terms such as sustained or vigilant attention, understood not only as a cognitive skill but also influenced by motivation, experiences and expectations (e.g., assumed rewards), cannot be equated with vigilance *per se*. In the view of test psychologists, vigilance is reflected by behaviors, and adequate reactions are not possible without appropriate stimulus perception. But Head's third aspect, the reorganizing function of vigilance, is not addressed by the usual sustained or vigilant alertness tasks; concepts other than a simple S-R model [e.g., dynamic self-regulation models (15)] may support this aspect but are not commonly in use.

## The Neurophysiological Concept of Vigilance

Findings in neurophysiology in the first half of the twentieth century proved to be largely supporting Head's concept of vigilance. For example, Hess (16) studies on the autonomic nervous system or, most importantly, the investigations of Bremer, Moruzzi and Magoun (17) on the ascending reticular activation system (ARAS). This pathway turned out to be significantly involved in maintaining wakefulness and alertness, as well as for short-term (phasic) and long-lasting (tonic) activation. Also in sleep, these neurobiological mechanisms are suspected to play a key role in sudden activations (arousals) of the cortex (18, 19). Although arousals show several similarities to Head's concept of vigilance, there are substantial differences. According to Head, vigilance describes a fundamental principle of living organisms rather than the performance of specific anatomical areas such as the ventrolateral preoptic nucleus, which is the case in arousals (20).

**Figure 1:**
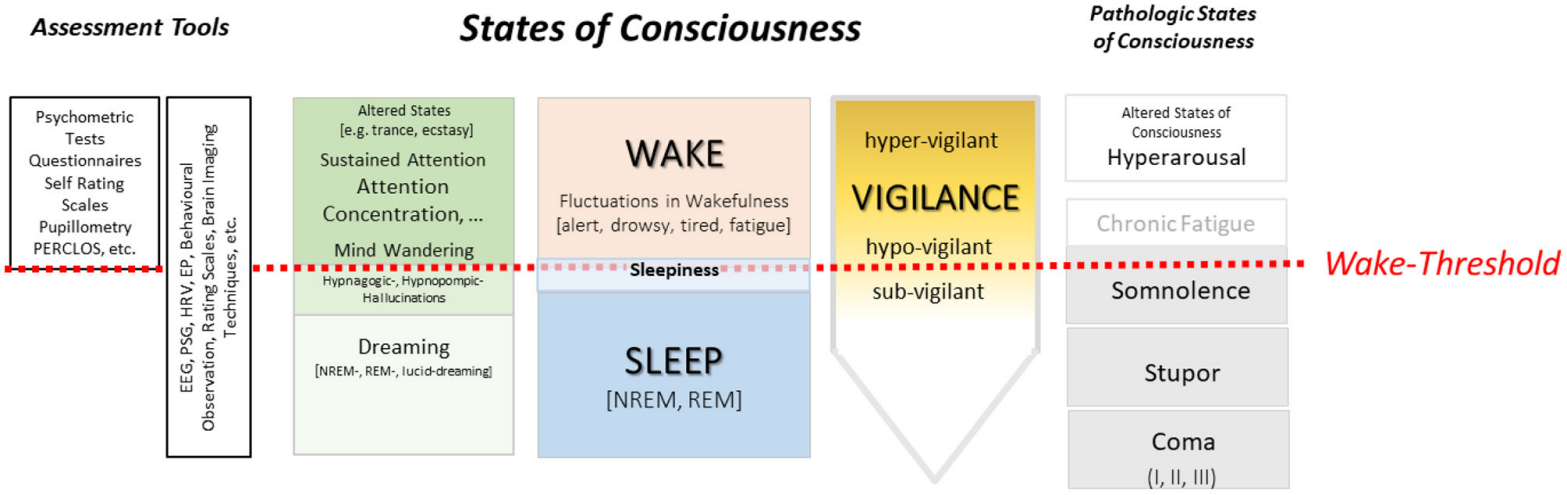
Overview of the different concepts and terminologies related to vigilance and states of consciousness (as well as pathologic states). In the graph also some wiedly used assessment tools are listed. For example, methods such electroencephalography (EEG), polysomnography (PSG), heart rate variability (HRV) or evoked potentials (EP) can be used for sleep and wake, whereas psychometric test or questionnaires are usable only in wake.

Technical innovations were another reason for the increasing interest in the neurophysiology of vigilance. At the end of the 1950s, the recording of brain activity utilizing multi-channel EEG systems was available in many neurobiological research units. Dieter Bente was among the first scientists in Europe creating a classification scheme of wake states, analogous to sleep stages. Attempts at classifying the waking state have existed since the late 1950s by Lindsley (20) and Roth (21). Fluctuations in wakefulness, visualized by flattening and slowing of the EEG-signals, were assigned to corresponding vigilance levels such as relaxed wakefulness (alpha waves; alert, vigilant), tense wakefulness (beta waves; active, overexcited, hypervigilant), or decreased alertness (alpha-theta waves; drowsy, hypo-vigilant) and “sub vigile stages” for the transition to sleep (22). The EEG was considered to be the ideal representative of vigilance because it enables the time-synchronous coupling of neuronal activity with observable behavior (23), which is not the case when solely using psychometric testing. However, there are different opinions on whether sleep stages should be included into the nomenclature of vigilance stages (24). The onset of sleep marks the boundary beyond which wakefulness definitely ends and in this context a semantic ambiguity becomes evident: in many EEG-studies, the term vigilance is synonymously used as wakefulness or even alertness and this vagueness is still evident (25). In addition, also other methodological issues dispatched [e.g., the concept of “local/global” vigilance (26)] and there is an ongoing controversy about the correct definitions of hyper-, hypo-, sub-, super– or supra-vigilance as compared to “normal” vigilance states (see Figure 1). Many of these concepts and definitions are deemed to be incompatible with Head's idea of vigilance as an integrative, non-divisible entity (2, 24).

To date, none of the classification schemes for wakefulness have gained acceptance (unlike sleep stages), neither in clinical medicine nor in basic research. As an alternative to define wake stages, a number of EEG-based vigilance indicators (27) such as the alpha slow wave index, the absolute delta power or the ‘vigilance index’ (28) have been proposed, but still, there is no consensus on which method is most suitable for measuring vigilance. Despite the fact that EEG studies are considered to be the gold standard for identifying fluctuations in vigilance, there are other psychometric methods (6, 29–32) which have proven to be sensitive and even more suitable for long term vigilance monitoring (33).

Head saw an important, if not the most essential function of vigilance in its reorganizing power. But this aspect was also not addressed in the neurophysiological discussion on vigilance. Bente (22) was one of the few who repeatedly pointed out the integrative function of vigilance and was convinced to determine the current level of neuronal organization (understood as an expression of vigilance) by the analysis of spontaneous brain electrical activity. This idea was picked up by Ulrich (2), a student of Bente, who defined vigilance in relation to a closed biological system which is in constant interaction with the environment. In this interplay of disorganization (i.e., partial opening of a closed biological system) and reorganization (i.e., functional development, restoration and system closure), vigilance could be the “force” behind these processes. Reframing the concept of vigilance with cybernetic and system-theoretical models (Norbert Wiener, Ludwig von Bertalanffy) could set new perspectives, especially with regard to the role of sleep as a homeostatic– and restorative process.

## Vigilance and the Sleep-Wake Cycle

Although some scientists consider sleep as a non-vigilant state and hence not a topic of interest, the transition from wake to sleep was intensively discussed in vigilance research. A wide range of hypotheses, classifications and assumptions were suggested to define the line between being awake, conscious, vigilant and asleep (24). In terms of sleep physiology, the process of falling asleep is clearly determined by visual polysomnographic criteria for sleep stages N1 and N2 (34), without further distinctions into sub-stages.

According to current opinion, the transition from wakefulness to sleep is not a succession of stages, but a continuous process, accompanied by highly selective deactivations of different brain areas. This is in line with the presumption that sleep may occur locally and not strictly as a global event (35, 36). In 1988, Koella (37) suggested a theoretical model for sleep/wake regulation, centered on a system which he called the “vigilance controlling apparatus” (VCA). In this model wake and sleep are not fundamentally opposite entities; they differ *phenomenologically* (37) only by their vigilance profiles (e.g., during relaxed wakefulness vigilance is at an intermediate level, whereas in sleep it is relatively low). The variability in these profiles depends on the level of local vigilance [another concept by Koella (26)], which is detectable by behavioral observation (e.g., the intensity, quality, precision and adequacy of behavior). Although Koella's considerations elicit only minor reactions in the sleep community, they are worth being reviewed, not only in the context of the “local/global sleep”-concept, but also in the discussion about default mode-networks (38, 39) and resting state phenomena (40, 41). This reorientation and re-definement of the concept vigilance in the context of sleep is crucial, because the term vigilance/vigilance states is often used interchangeably with sleep stages, sleepiness or wakefulness (25).

In the diagnosis of sleep disorders, vigilance tasks are important to point out the consequences of poor sleep on daytime sleepiness, fatigue or tiredness (42). Besides psychometric tasks such as the PVT or simple reaction time tests also the MSLT, MWT or other psychophysiological measurements (e.g., heart-rate, actigraphy, evoked potential, pupillography) are in use (6) (see Table 1). The diagnostic value of such procedures does not always justify the great effort behind some of these tests (57). Moreover, the correlation between the different tasks is rather poor (58), particularly in patients whose subjective ratings of fatigue and tiredness rarely fit with objective measurements (59, 60).

**Table 1:** Overview of the different definitions of vigilance, attention (vigilant, sustained), alertness, sleepiness, fatigue, tiredness and related tasks paradigms (measurements).

	**Definitions**	**Measurements** **(not a complete overview)**
**vigilance**	- ability to reorganize and restore damaged functions in order to react adequately to environmental stimuli (1) - degree of central nervous activation: • high = hypervigilant (high degree of activartion can lead to a lack of reaction • low = hypovigilant: subject cannot adequately react to external stimuli under monotonous conditions (6) - capability to be aware of potential relevant, unpredictable changes in one's environment, including a quantitative dimension, a sufficient level of alertness, and a temporal dimension (43)	- ‘conventional’ vigilance tests: monotonuous, long lasting, with infrequently ap-pearing target stimuli [e.g., Mackworth Clock Tests (3)] - alternatively: short tasks, but with more target stimuli (e.g., Psychomotor Vigilance Test (PVT) (8), reaction time tasks (RTT), go/no-go tasks, forced choise tasks (FCT) - electroencephalography (EEG), polysomnography (PSG), evoked potential (EP) - heart rate variability (HRV) - pupillometry - electrodermal activity (EDA) - functional magnetic resonance imaging (fMRI) - videometry
**attention**	ability to watch, listen to, concentrate, or to focus one's mind on some-thing/someone with interest (requires cognitive control)	- psychometric tests for attention, concentration [e.g., Attention Network Test (44)], go/no-go tasks, FCT - EEG, EP, fMRI
**vigilant attention, sustained attention**	ability to maintain focused and stable across long time intervals (45); the decline in timely and correct responses is defined as vigilance decrement or time-on-task effects	- Mackworth Clock Test, PVT, RTT, FCT, go/no-go tasks - EEG, PSG, HRV, fMRI
**alertness**	state of being awake, prepared to act/react; also defined as the result of the interplay between circadian processes, sleep-homeostasis and sleep inertia (46); influenced by time-awake-, time-on-duty or time-on-task	- PVT, RTT, go/no-go tasks, FCT - EEG, PSG, EP, HRV
**sleepiness**	subjective expression of the individuals need of sleep (feeling of being sleepy); sleepiness characterizes the transition between being alert (awake, fully awake) and falling asleep, accompanied by subjective (cognitive) and objective (physiological, behavioral) changes	subjective (cognitive) measurements: Karolinska Sleepiness Scale (KSS) (47), Stanford Sleepiness Test (SSS) (48), Epworth Sleepiness Scale (ESS) (49) objective (physiological) measurements: Multiple Sleep Latency Test (MSLT) (50), Maintenance of Wakefulness Test (MWT) (51), pupillometry, videometry
**fatigue, tiredness**	*fatigue* = subjective sense of tiredness; influenced by two biological factors: sleep-homeostasis and circadian processes; depends on the time-awake, time-on-duty or time-on-task; “fatigue” is often interchangeable used with sleepiness or tiredness; therefore it is important to distinguish between mental and physical fatigue (52). *tiredness* = diurnal fluctuations of wakefulness, contrary to sleepiness because (daytime) sleepiness is a sign of non-restorative sleep (53).	subjective (cognitive) measurements: e.g., Fatigue Severity Scale (FSS) (54), Fatigue Assessment Instrument (FAI) (55) objective (physiological) measurements: e.g., PVT, RTT, FCT, go/no-go tasks PERCLOS (56), EEG, PSG, EP, videometry

One reason for these shortcomings is the lacking comprehensive concept of vigilance in basic sleep research; an attempt at such a concept by Posner and Rafal (the attention model) has yet to be updated (61). Approaches with attention triggered by cues, inwards (e.g., linked to mind wandering and daydreams) or outwards orientated (directed or selective) or reinforcing behaviors over time (as a sign of vigilance) may foster new insights as well as discussing the role of awareness in the context of vigilance (62). Currently, vigilance testing in sleep medicine is characterized by a cocktail of test methods (6, 30, 42) and confusing definitions taken over from neighboring disciplines, particularly psychology (see Table 1). New insights may offer functional magnetic resonance imaging studies with sleep deprived subjects (25, 63, 64).

## Revisiting the Concept of Vigilance

It is certainly not necessary to reinvent the concept of vigilance; A look at the extensive literature on this topic proves that there are already enough concepts and ideas. Looking back and anticipating Head's genuine considerations is enough to gather new ideas and perspectives. As a first step, it is necessary to clearly distinguish vigilance from other concepts such as alertness, attention or arousal (43). By doing this, we suggest a second step to reframe the concept of vigilance as a mindset for collecting data on sleep-wake behaviors (e.g., psychometric testing, sleep studies, behavioral observation, subjective data, etc.) and their interpretation (see Figure 1). Research on vigilance should consider three *perspectives*:

### Vigilance—Allocation of Resources

From a neurophysiological point of view, tiredness, fatigue, and decrements in attention and concentration are the consequences of neuronal inhibition, habituation or, more generally speaking, the decrease of alertness-promoting compounds (monoamines, acetylcholine). Subsequently, substances inhibiting neuronal signal processing accumulate [e.g., adenosine (65)] and thus produce “sleep pressure”. We consider wakefulness and alertness as biological resources which guarantee adequate reactions, ultimately for survival. If these resources tail off, compensatory actions are initiated, which are observable (e.g., behavioral patterns such as stretching, yawning) and measurable. In our opinion, the process of allocating resources is a matter of vigilance.

### Vigilance—Compensatory Behaviors

Sleepy subjects try to keep themselves awake through auto-stimulating behavior (yawning, stretching, lolling, singing, whistling) (66, 67). These subsidiary behaviors (68, 69) are an expression of compensatory mechanisms to replace or mitigate diminishing alertness in order to fight against falling asleep. For example, children sometimes show hyperactive behavior at bedtime as a countermeasure for sleepiness. We think that compensatory actions such as subsidiary behaviors are a sign of the vigilant subject and thus relates to Head's 3rd sub-service of vigilance (*reorganization*). Therefore, the identification and documentation of compensatory or subsidiary behaviors should be an essential part of vigilance diagnostics. With the use of video recordings and image processing tools, behavioral observation is feasible without consuming too much time and guarantees objective data analysis (66, 67, 70, 71).

### Vigilance—(Human) Environmental Interactions

Many situations in daily life necessitate increased levels of attention and concentration. Activities like driving on the motorway for several hours at night, paying attention in class, or simply crossing a busy road require a substantial level of attention, not only to the environment, but also to one's ‘inner’ world (i.e., emotions, motivation). The interplay between self-perception (e.g., ‘I'm sleepy because I didn't sleep the night before’) and environmental demands (e.g., the consequences of errors) produce tension and activation, which are not considered by conventional vigilance test settings. For example, studies on driver fatigue demonstrate that car driving simulators do not reflect the situations of driving on a road at night. In real life, drivers are less tired and sleepy as compared to experimental settings in a lab (72). Therefore, we suggest including information about the test setting in clinical practice as well as in basic scientific research.

## Conclusions and Further Perspectives

Good sleep has numerous effects such as recovery from physical and emotional stress, and being well-rested, alert, concentrated, and productive during wakefulness. Vigilance is essential to guarantees adequate reactions to any kind of stimuli in order to ensure adaptation to changing environmental conditions. Conceived as a theoretical model (or construct), vigilance can be indirectly measured through psychophysiological methods or observed through *visible behavioral cues*, in particular by the degree (e.g., intensity, speed etc.) of ordered reactions including automatic behaviors. In our opinion, behavioral observation plays a key role in vigilance monitoring. Alertness, awareness or attentiveness are vigilance-associated processes, but not equivalents of vigilance. We suggest three new directions in future research on vigilance: 1st the role of vigilance in allocating resources (as a conceptual and explanatory mindset), 2nd vigilance as a trigger for subsidiary behaviors (“measured” by behavioral observations), and 3rd vigilance embedded in environmental interactions (which consider information about the test setting to be essential for classifying vigilant behaviors). Some of these suggestions have already been implemented with promising results (66, 67, 70, 71, 73).

Vigilance, re-defined as a system of allocation and reorganization of biological resources, provides a better understanding of sleep-wake behaviors and allows for the consequences of non-restorative sleep to be assessed in more detail. This approach may also improve the validity of bio-mathematical models in fatigue risk management to predict fatigue-related decrements in performance (74).

## Data Availability Statement

The original contributions presented in the study are included in the article/supplementary material, further inquiries can be directed to the corresponding author/s.

## Author Contributions

GK: conceptualization and wirting—draft manuscript. JZ: manuscript editing and review. OI: conceptualization and manuscript editing. All authors contributed to the article and approved the submitted version.

## Conflict of Interest

The authors declare that the research was conducted in the absence of any commercial or financial relationships that could be construed as a potential conflict of interest.

## Publisher's Note

All claims expressed in this article are solely those of the authors and do not necessarily represent those of their affiliated organizations, or those of the publisher, the editors and the reviewers. Any product that may be evaluated in this article, or claim that may be made by its manufacturer, is not guaranteed or endorsed by the publisher.

## References

[1] Head H. 'Vigilance'; a physiological state of the nervous system. Brit J Psycho Gen Sect. (1923) 14:126–47. 10.1111/j.2044-8295.1923.tb00122.x

[2] Ulrich G. The importance of the concept vigilance for psychophysiological research. Med Hypotheses. (1988) 27:227–9. 10.1016/0306-9877(88)90149-1 3211021

[3] Mackworth NH. The breakdown of vigilance during prolonged visual search. Quart J Exp Psychol. (1948) 1:6–21. 10.1080/17470214808416738

[4] Buckner DN McGrath JJ. Vigilance: a Symposium. (1963). New York, San Francisco, Toronto, London: McGraw-Hill Book Company.

[5] Stroh CM. Vigilance: The Problem of Sustained Attention. (1971). Oxford, New York, Toronto, Sydney, Braunschweig: Pergamon Press.

[6] Canisius S Penzel T. Vigilance monitoring—review and practical aspects. Biomed Tech. (2007) 52:77–82. 10.1515/BMT.2007.015 17313339

[7] Wilkinson RT Houghton D. Field test of arousal: a portable reaction timer with data storage. Hum Factors. (1982) 24:487–93. 10.1177/001872088202400409 7129455

[8] Dinges DF Powell JW. Microcomputer analyses of performance on a portable, simple visual RT task during sustained operations. Behav Res Meth Instr Comp. (1985) 17:652–5. 10.3758/BF03200977

[9] Oken BS Salinsky MC Elsas SM. Vigilance, alertness, or sustained attention: physiological basis and measurement. Clin Neurophysiol. (2006) 117:1885–901. 10.1016/j.clinph.2006.01.017 16581292 PMC2865224

[10] Hudson AN Van Dongen HPA Horn K. Sleep deprivation, vigilant attention, and brain function: a review. Neuropsychopharmacology. (2020) 45:21–30. 10.1038/s41386-019-0432-6 31176308 PMC6879580

[11] Weitkunat R Bestler M. Computerized Mackworth vigilance clock test. Comput Meth Prog Biomed. (1990) 32:147–9. 10.1016/0169-2607(90)90095-Q 2397639

[12] Lichstein KL Riedel BW Richman SL. The Mackworth Clock Test: a computerized version. J Psychol. (2000) 134:153–61. 10.1080/00223980009600858 10766107

[13] Lim J Dinges DF. Sleep deprivation and vigilant attention. Ann NY Acad Sci. (2008) 1129:305–22. 10.1196/annals.1417.002 18591490

[14] Doran S Van Dongen HPA Dinges D. Sustained attention performance during sleep deprivation: evidence of state instability. Arch Ital Biol. (2001) 139:253–67. 10.4449/aib.v139i3.503 11330205

[15] Karoly P. Mechanisms of self-regulation: a systems view. Annu Rev Psychol. (1993) 44:23–52. 10.1146/annurev.ps.44.020193.000323

[16] Hess WR. Über die Wechselbeziehung zwischen psychischen und vegetativen Funktionen. Schweiz Arch Neurol Psych. (1925) 15:260–77.

[17] Moruzzi G Magoun HW. Brain stem reticular formation and activation of the EEG. Electroencephalogr Clin Neurophysiol. (1949) 1:455–73. 10.1016/0013-4694(49)90219-9 18421835

[18] Monroe LJ. Psychological and physiological differences between good and poor sleepers. J Abnormal Psychol. (1967) 72:255–64. 10.1037/h0024563 6045597

[19] Riemann D Spiegelhalder K Feige B Voderholzer U Berger M Perlis M. The hyperarousal model of insomnia: a review of the concept and its evidence. Sleep Med Rev. (2010) 14:19–31. 10.1016/j.smrv.2009.04.002 19481481

[20] Lindsley DB. Attention, consciousness, sleep & wakefulness. In: Field J Magoun HW Hall VE. (Eds). Handbook of Psychophysiology, Section I, Neurophysiology (Vol II). (1960). Washington DC: American Physiological Society.

[21] Roth B. The clinical and theoretical importance of EEG rhythms corresponding to state lowered vigilance. Electroencephalogr Clin Neurophysiol. (1961) 13:395–9. 10.1016/0013-4694(61)90008-6 14494365

[22] Bente D. Die Insuffizienz des Vigilanztonus. (1964). Erlangen: Habilitationsschrift, Müller Verlag.

[23] Streitberg B Röhmel J Herrmann WM Kubicki S. COMSTAT rule for vigilance classification based on spontaneous EEG activity. Neuropsychobiology. (1987) 17:105–17. 10.1159/000118347 3627388

[24] Kugler J Leutner V. Vigilanz. Ihre Bestimmung und Beeinflussung. (1984). Basel: Edition Roche.

[25] Czisch M Wehrle R Harsay HA Wetter TC Holsboer F Sämann P. On the need of objective vigilance monitoring: effects of sleep loss on target detection and task-negative activity using combined EEG/fMRI. Front Neurol. (2012) 3:67. 10.3389/fneur.2012.00067 22557992 PMC3338067

[26] Koella WP. Vigilance—local vigilance—the vigilance profile: a new concept and its application in neurobiology and biological psychiatry. Acta Neurol Scand. (1984) 99: 35–41. 10.1111/j.1600-0404.1984.tb05667.x 6146238

[27] Sander C Hensch T Wittekind DA Böttger D Hegerl U. Assessment of wakefulness and brain arousal regulation in psychiatric research. Neuropsychobiology. (2015) 72:195–205. 10.1159/000439384 26901462

[28] Herrmann WM Kern U Röhmel J. Contribution to the search for vigilance-indicative EEG variables. Results of a controlled, double-blind study with pyritinol in elderly patients with symptoms of mental dysfunction. Pharmacopsychiatry. (1986) 19:75–83. 10.1055/s-2007-1017159 3517890

[29] Raymann R van Someren E. Time-on-task impairment of psychomotor vigilance is affected by mild skin warming and changes with aging and insomnia. Sleep. (2007) 30:96–103. 10.1093/sleep/30.1.96 17310870

[30] Maire M Reichert CF Gabel V Viola AU Phillips C Berthomier C. Human brain patterns underlying vigilant attention: impact of sleep debt, circadian phase and attentional engagement. Sci Rep. (2018) 8:970. 10.1038/s41598-017-17022-9 29343686 PMC5772468

[31] Haubert A Walsh M Boyd R Morris M Wiedbusch M Krusmark M. Relationship of event-related potentials to the vigilance decrement. Front Psychol. (2018) 9:237. 10.3389/fpsyg.2018.00237 29559936 PMC5845631

[32] Maccora J Manousakis JE Anderson C. Pupillary instability as an accurate, objective marker of alertness failure and performance impairment. J Sleep Res e. (2018). 10.1111/jsr.12739 30062813

[33] Henelius A Sallinen M Huotilainen M Müller K Virkkala J Puolamäki K. Heart rate variability for evaluating vigilant attention in partial chronic sleep restriction. Sleep. (2014) 37:1257–67. 10.5665/sleep.3850 24987165 PMC4074968

[34] Iber C Ancoli-Israel Chesson A Quan SF for the American Academy of Sleep Medicine. The AASM Manual for the Scoring of Sleep and Associated Events: Rules, Terminology and Technical Specifications. (2007). Westchester, Illinois: American Academy of Sleep Medicine.

[35] Siclari F Tononi G. Local aspects of sleep and wakefulness. Curr Opin Neurobiol. (2017) 44:222–7. 10.1016/j.conb.2017.05.008 28575720 PMC6445546

[36] Huber R Ghilardi MF Massimini M Tononi G. Local sleep and learning. Nature. (2004) 430:78–81. 10.1038/nature02663 15184907

[37] Koella WP. The organization and regulation of sleep. Experientia. (1984) 40:309–408. 10.1007/BF01952538 6370714

[38] Hinds O Thompson TW Ghosh S Yoo JJ Whitefield-Gabrieli S Triantafyllou C. Roles of default-mode network and supplementary motor area in human vigilance performance: evidence from real-time fMRI. J Neurophysiol. (2013) 109:1250–8. 10.1152/jn.00533.2011 23236006

[39] Bogler C Vowinkel A Zhutovsky P Haynes JD. Default network activity is associated with better performance in a vigilance task. Front Hum Neurosci. (2017) 11:623. 10.3389/fnhum.2017.00623 29311878 PMC5743927

[40] Raichle ME. The brain's default mode network. Annu Rev Neurosci. (2016) 38:433–47. 10.1146/annurev-neuro-071013-014030 25938726

[41] Falahpour M Chang C Wong CW Liu T. Template-based prediction of vigilance fluctuations in resting-state fMRI. Neuroimage. (2018) 174:317–27. 10.1016/j.neuroimage.2018.03.012 29548849 PMC8328148

[42] Shahid A Shen J Shapiro CM. Measurements of sleepiness and fatigue. J Psychosom Res. (2010) 69:81–9. 10.1016/j.jpsychores.2010.04.001 20630266

[43] Van Schie MKM Lammers GJ Fronczek R Middelkoop HAM van Dijk JG. Vigilance: discussion of related concepts and proposal for a definition. Sleep Med. (2021) 83:175–81. 10.1016/j.sleep.2021.04.038 34022494

[44] Fan J McCansliss BD Fossella J Flombaum JI Posner MI. The activation of attentional networks. Neuroimage. (2005) 26:471–9. 10.1016/j.neuroimage.2005.02.004 15907304

[45] Warm J Parasuraman R Mathews G. Vigilance requires mental work and is stressful. Hum Factors. (2008) 50:433–41. 10.1518/001872008X312152 18689050

[46] Ǻkerstedt T Folkard S. Predicting duration of sleep from the three process model of regulation of alertness. Occup Environ Med. (1996) 53:136–41. 10.1136/oem.53.2.136 8777451 PMC1128427

[47] Ǻkerstedt T Gillberg M. Subjective and objective sleepiness in the active individual. Int J Neurosci. (1990) 52:29–37. 10.3109/00207459008994241 2265922

[48] Hoddes E Zarcone V Smythe H Phillips R Dement WC. Quantification of sleepiness: a new approach. Psychophysiology. (1973) 10:431–7. 10.1111/j.1469-8986.1973.tb00801.x 4719486

[49] Johns MW. A new method for measuring daytime sleepiness: the Epworth Sleepiness Scale. Sleep. (1991) 14:540–5. 10.1093/sleep/14.6.540 1798888

[50] Carskadon MA Dement WC Mitler MM Roth T Westbrook PR Keenan S. Guidelines fort he multiple sleep latency test (MSLT): a standard measure of sleepiness. Sleep. (1986) 9:519–24. 10.1093/sleep/9.4.519 3809866

[51] Doghramji K Mitler M Sangal RB Shapiro C Taylor S Walsleben J. A normative study of the Maintenance Of Wakefulness Test (MWT). Electroencehalogr Clin Neurophysiol. (1997) 103:554–62. 10.1016/S0013-4694(97)00010-2 9402886 PMC2424234

[52] Mehta RK Parasuraman R. Effects of mental fatigue on the development of physical fatigue: a neuroergonomic approach. Hum Factors. (2014) 56:645–56. 10.1177/0018720813507279 25029891

[53] Weeß HG Sauter C Geisler P Böhning W Wilhelm B Rotte M. Vigilance, tendency to fall asleep, sustained attention, tiredness, sleepiness – diagnostic tools fort he measurement of sleepiness related processes and their criteria of quality. Somnologie. (2000) 4:20–38. 10.1046/j.1439-054x.2000.00116.x

[54] Krupp LB LaRocca NG Muir-Nash J Steinberg AD. The fatigue severity scale. Application to patients with multiple sclerosis and systemic lupus erythematosus. Arch Neurol. (1989) 46:1121–3. 10.1001/archneur.1989.00520460115022 2803071

[55] Schwartz JE Jandorf L Krupp LB. The measurement of fatigue: a new instrument. J Psychosom Res. (1993) 37:753–62. 10.1016/0022-3999(93)90104-N 8229906

[56] Dinges D Grace R. PERCLOS: A Valid Psychophysiological Measure of Alertness as Assessed by Psychomotor Vigilance. (1998). TechBrief NHTSA, Publication No. FHWA-MCRT-98-006

[57] Arand DL Bonnet M. The multiple sleep latency test. Handb Clin Neurol. (2019) 160:393–403. 10.1016/B978-0-444-64032-1.00026-6 31277864

[58] Baiardi S Mondini S. Inside the clinical evaluation of sleepiness: subjective and objective tools. Sleep Breath. (2020) 24:369–77. 10.1007/s11325-019-01866-8 31144154

[59] Sangal RB Sangal MJ Belisle C. Subjective and objective indices of sleepiness (ESS and MWT) are not equally useful in patients with sleep apnea. Clin Electroencephalogr. (1999) 30:73–5. 10.1177/155005949903000208 10358786

[60] Trimmel K Eder HG Böck M Stefanic-Kejik A Klösch G Seidel St. The (mis)perception of sleep: factors influencing the discrepancy between self-reported and objective sleep parameters. J Clin Sleep Med. (2021) 17:917–24. 10.5664/jcsm.9086 33393901 PMC8320481

[61] Posner M Rafal R. Cognitive theories of attention and the rehabilitation of attentional deficits. 182–201. In: Meier M Benton A Diller L (Eds): Neuropsychological Rehabilitation. (1987). Churchill Livingstone, Edinburgh.

[62] Craig BAD. How do you feel – now? The anterior insular and human awareness. Nat Rev Neurosci. (2009) 10:59–70. 10.1038/nrn2555 19096369

[63] Maniscalco B McCurdy L Odegaard B Lau H. Limited cognitive resources explain a trade-off between perceptual and metacognitive vigilance. J Neurosci. (2017) 37:1213–24. 10.1523/JNEUROSCI.2271-13.2016 28028197 PMC6596853

[64] Teng J Ong JL Patanaik A Tandi J Zhou JH Chee M. Vigilance declines following sleep deprivation are associated with two previously identified dynamic connectivity states. Neuroimage. (2019) 200:382–90. 10.1016/j.neuroimage.2019.07.004 31276798

[65] Porkka-Heiskanen T Zitting KM Wigren HK. Sleep, its regulation and possible mechanism of sleep disturbances. Acta Physiol. (2013) 208:311–28. 10.1111/apha.12134 23746394

[66] Kemethofer M. Behavior Patterns and Effects of Napping During Night-Time Driving. (2013). Master thesis online http://othes.univie.ac.at/27554/

[67] Tse E Bao S Campbell M Carson N Hussaina H Maher KS. Behavioural observations step 3: vigilance of night-time drivers. Sleep Med. (2017) 40:e3–185. 10.1016/j.sleep.2017.11.483

[68] Rogé J Pebayle T Muzet A. Variations of the level of vigilance and of behavioral activities during simulated automobile driving. Accid Anal Prev. (2001) 33:181–6. 10.1016/S0001-4575(00)00029-4 11204888

[69] Takanishi T Ebara T Murasaki G Kubo T Tachi N Itani T. Interactive model of subsidiary behaviors, work performance and autonomic nerve activity during visual display terminal work. J Occup Health. (2010) 52:39–47. 10.1539/joh.L9110 20032590

[70] Ipsiroglu OS Klösch G Beyzaei N McCabe S Berger M Kühle HJ. Video recordings of naturalistic observations: pattern recognition of disruptive behaviors in people with mental health or neurodevelopmental conditions. (54–82) In: Kerzel S Paditz E. (Eds). Aktuelle Kinderschlafmedizin, kleanthes, Dresden (2017)

[71] Coronel C Wiesmeyr C Garn H Kohn B Wimmer M Mandl M. 3D camera and pulse oximeter for respiratory event detection. IEEE J Biomed Health Inform. (2021) 25:181–8. 10.1109/JBHI.2020.2984954 32324578

[72] Hallvig D Anund A Fors C Kecklund G Karlsson JG Wahde M. Sleepy driving on the real road and in the simulator – a comparison. Accid Anal Prev. (2012) 50:44–50. 10.1016/j.aap.2012.09.033 23149323

[73] Ipsiroglu OS Hung Y Chan F Ross M Veer D Soo S. ‘Diagnosis by behavioral observation’ home-videosomnography—a rigorous ethnographic approach to sleep of children with neurodevelopmental conditions. Front Psychiatry. (2015) 6:39. 10.3389/fpsyt.2015.00039 25852578 PMC4362082

[74] Dawson D Darwent D Roach GD. How should a bio-mathematical model be used within a fatigue risk management system to determine whether or not a working time arrangement is safe? Accid Anal Prev. (2017) 99:469–73. 10.1016/j.aap.2015.11.032 27040118

